# *P* element activity and molecular structure in *Drosophila melanogaster* populations from Firtina Valley, Turkey

**DOI:** 10.1093/jis/14.1.16

**Published:** 2014-01-01

**Authors:** Banu Sebnem Onder, Ozge Erisöz Kasap

**Affiliations:** Hacettepe University, Faculty of Science, Biology Department, 06800 Beytepe-Ankara, Turkey

**Keywords:** geoclimatic variables, transposable elements

## Abstract

In order to study *P* element dynamics in natural populations of *Drosophila melanogaster* , 88 isofemale lines were examined from the Firtina Valley, Turkey. The P-M gonadal dysgenesis characteristics and the molecular patterns of *P* and *KP* elements were analyzed. Gonadal dysgenesis tests showed a slight variation both for *P* activity and *P* susceptibility, however the results showed a predominant M' phenotype for this region. The *P* and *KP* element were also characterized by polymerase chain reaction. The molecular analyses showed that all the populations examined had the entire 1.15 kb *KP* element. The molecular patterns of *KP* elements were the same for the populations studied. No clear relationship was found between phenotype and genomic *P* element composition. The correlations between the level of gonadal dysgenesis percentage (as an index for *P* activity and *P* susceptibility) and several geoclimatic factors were tested, and no general effects of altitude, temperature, rainfall, or humidity were found. The theoretical P' strain, which is very rare in natural populations, was also recorded for this region.

## Introduction

The most well-known transposable element in natural populations of *Drosophila melanogaster* is the *P* element. *P* elements have been classified into two structural types, the 2907 base pair (bp) long complete elements and the smaller elements with internal deletions. Smaller elements with deletions involving intron 3 are called type I, and *P* elements that have larger deletions are called type II elements. The *KP* element, which originates from full-size *P* element by an internal deletion (808-2560), is one of the most common type II elements in natural populations ( Black et al. 1987 ; Jackson et al. 1988 ; Itoh et al. 2001 , 2002, 2004).The complete *P* elements encode an 87 kDa transposase and a 66 kDa repressor protein. *P* element transposition is controlled by these proteins, thus the complete *P* elements are autonomous ( O’Hare and Rubin 1983 ). The transposase is only synthesized in the germ line cells, and *P* element transposition is limited to the germ line ( Karess and Rubin 1984 ; Laski et al. 1986 ; Rio et al. 1986 ). Type I *P* elements can produce only the 66 kDa repressor protein or its equivalent ( Gloor et al. 1993 ). Type II elements cannot produce the transposase or repressor protein, but are capable of repressing *P* transposition because of the short proteins they encode (Andrew and Gloor 1995). If all these elements maintain the inverted terminal repeats, they can be mobile in the presence of the transposase protein from a 2,907 bp element.

*P* elements are a causative factor for P-M hybrid dysgenesis in *D. melanogaster* ( Kidwell et al. 1977 ; Engels 1996 ). The crossing between males of a strain containing *P* elements (P strain) and females lacking *P* elements (M strain) causes a transposition of *P* elements in the germline cells, which results in sterility by gonadal dysgenesis (GD), chromosomal breaks or rearrangements, mutations, and male recombination ( Kidwell et al. 1977 ; Engels and Preston 1980 ; Engels 1989 , 1996). The effects occurred maternally, because no dysgenic effects were observed in the reciprocal cross ( Kidwell et al. 1977 ; Engels 1979 ). Strains are classified into five phenotypes according to the ability to induce ( *P* activity potential) or to repress ( *P* susceptibility) *P* transposition: the P strain (strong *P* activity and low level of *P* susceptibility), Q strain (low level of *P* activity and susceptibility), M' strain (low level of *P* activity and strong *P* susceptibility) and M strain (without *P* activity and strong *P* susceptibility), and the exceptional P' strain (strong *P* activity and *P* susceptibility) ( Kidwell et al. 1977 ; Engels and Preston 1980 ; Quesneville and Anxolabéhère 1998 ). M strains do not have the autonomous and non-autonomous *P* elements and their derivatives. Furthermore, if an M strain is carrying some *P* sequences in the genome it is called M' strain ( Bingham et al. 1982 ).

*P* element was inserted in the genome of *D. melanogaster* by recent horizontal transfer from another *Drosophila* species ( Kidwell 1983 ; Anxolabéhère et al. 1988 ; Houck et al. 1991 ). It has rapidly spread into the wild type *D. melanogaster* populations all around the world. The currently common and predominant P-M phenotype in nature is the M' type. The M' strains are predominant in Asia, Europe, North Africa, and Southeast Australia ( Anxolabéhère et al. 1984 , 1985; Kidwell 1983 ; Boussy et al. 1988 ). Most of these M' strains have the derivative *KP* element in their genome. Copies belonging to the *P* elements have been found in all natural populations of *D. melanogaster* tested. True M strains have not been found more recently than a strain collected in the former city of Gorky, Russia (now Nizhny Novgorod) in 1974 (Anxolabéhère et al. 1988).

*D. melanogaster* populations show different frequencies of full-sized and defective *P* elements. The P-M phenotypes vary worldwide in natural *D. melanogaster* populations. Weak correlation was found in many natural populations between the genomic *P* element and the phenotypes (Anxolabehere et al. 1985, 1988, 1990; Boussy and Kidwell 1987 ; Boussy et al. 1988 , 1998; Biemont et al. 1990; Ronsseray et al. 1991 ; Bonnivard and Higuet 1999 ; Itoh et al. 1999 , 2001, 2004, 2007; Itoh and Boussy 2002 ; Onder and Bozcuk 2012 ). In this scope it is known that the activity is affected by the genetic composition and the position of *P* element in the genome. Transposable element activities may also be induced by environmental factors ( Capy et al. 2000 ). Environmental factors affect the activity of *P* element, and the induced effect of high temperature on the activity of *P* element is quite common. Ruiz and Carareto (2003) proposed that the *P* element copy number is under the influence of the temperature of the original location of each population. This association between temperature and *P* element might have a selective effect on other ecological variables correlated with temperature, such as food resources, the presence of competitors, predators, or pathogens, or other unknown variables (Gonzalez et al. 2010). Several studies refer to geographical clusters of P-M phenotypes ( Boussy and Kidwell 1987 ; Bonnivard and Higuet 1999; Ruiz and Carareto 2003 ; Onder and Bozuck 2012 ). However, the effect of environmental factors on the *P* element activity and susceptibility of these geographically distinct natural populations are less known. Most of these factors have been discussed from a theoretical point of view, and very few data are available for natural populations.

A previous study gave a general view of P-M phenotypes in several Turkish populations of *D. melanogaster* and their relationships with climatic and geographic variables, pointing out an interaction between *P* susceptibility and longitude and a relationship between *P* activity and rainfall ( Onder and Bozcuk 2012 ). Could an environmental stress factor such as rainfall lead to an increase of the *P* activity? Is there a correlation between stressful conditions such as rainfall, humidity, or temperature and *P* activity or susceptibility? To answer these questions, wild *D. melanogaster* strains from the rainy Black Sea region in Turkey, the Firtina Valley, were examined.

## Materials and Methods

### Flies

To avoid the effects of local weather patterns of Firtina Valley, one sampling locality was chosen near the coastal area and the other four localities (V1–4) were chosen along a transect in the valley located at different altitudinal levels. Populations from the different localities were represented as samplings of isofemale lines (each line started from a single wild-caught female), which were collected using banana traps in August 2009. The localities, number of tested lines, and details of the collections are given in Table 1 . Flies were maintained on standard corn-meal food medium at 21° C and 55% RH for laboratory rearing, and GD tests were performed using the third generation flies at 29° C as described in detail below.

**Table 1.**
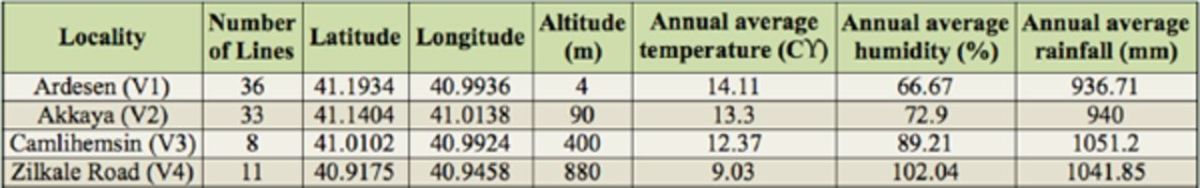
Sampling localities from which isofemale lines were collected.

### Sampling area

**Figure 2.**
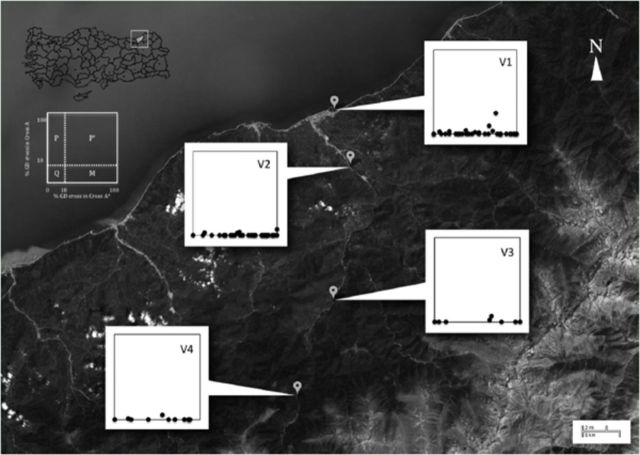
The results of GD tests for populations from Firtina Valley. Collection sites: V1: Ardesen, V2: Akkaya, V3: Camlihemsin, and V4: Zilkale Road. The graphs represent the GD percentage for the populations in cross A (vertical axis) and cross A* (horizontal axis). Each dot represents data for an isofemale line. Definition graph points out the P-M system according to Kidwell (1983) and Quesneville and Anxolabéhère (1998) . High quality figures are available online.

Samplings were conducted from the coast to the Firtina Valley along different altitudes ( Figure 2 ). The valley was covered with warm-deciduous forests existing without interruption ( Saglam and Caglar 2005 , 2007) and was surrounded by dense tree lines reaching over 30 meters. The tree lines isolated the valley from wind currents on all sides, therefore wind activity within the valley was highly reduced. The valley receives abundant rainfall all year long, with mean precipitation values reaching 1296.5 mm. Temperatures are usually low, with a yearly mean of 13.5° C. The highest temperatures are recorded in July and August, with mean values around 21.7° C, but temperatures within the day can vary depending on sunlight and rainfall. Relative humidity is high and constant throughout the year, with mean values around 73–82% RH ( Saglam and Caglar 2005 , 2007). To quantify the micro-climatic conditions of each collection site ( Table 1 ), daily temperatures and relative humidity were measured using data loggers (iButton hygrochron, DS 1923, Maxim Integrated, www.maximintegrated.com ). Rainfall data were obtained from the Turkish State Meteorological Service from the nearest weather station to the original capture site and from the Davis Vantage model weather station set in Camlihemsin (V3).

### Gonadal dysgenesis tests

With Harwich and Canton-S as P and M line standards, respectively, two kinds of crosses, A (Canton-S females x tested males) and A* (tested females x Harwich males), were performed at 29° C ( Kidwell et al. 1977 ; Engels and Preston 1980 ). Three crosses were routinely made for each isofemale line, more than 35 F _1_ females were dissected for each cross, and the GD score for each line was calculated as the percentage of undeveloped ovaries. At the same time, Harwich males were crossed with Canton-S females as a control, which resulted in 100% dysgenic ovaries. P-M characteristics were defined according to Kidwell (1983) : P strain (> 10% GD in cross A and < 10% GD in cross A*), Q strain (< 10% GD in both crosses A and A*), M' strain ( < 10% GD in cross A and >10% GD in cross A*), M strain (0% in cross A and 100% GD in cross A*). Exceptional P' strain (> 10% in both crosses A and A*) was defined according to Quesneville and Anxolabéhère (1998) . In addition, the M' strain was an M line with *P* sequences in the genome ( Bingham et al. 1982 ).

### Genomic DNA isolation and PCR

**Figure 1.**
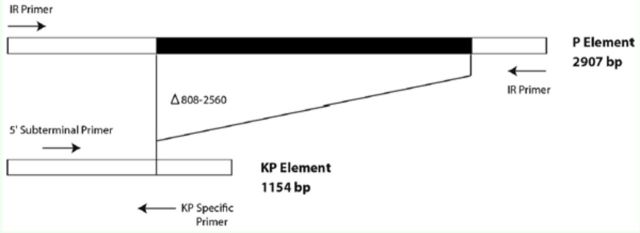
Schematic drawing of the 2.9 kb full sized *P* element and the 1.15 kb *KP* element. The deletion endpoints of the *KP* element and all primer pair binding sites used in the PCR analyses are shown. High quality figures are available online.

A total of 88 isofemale lines of *D. melanogaster* were studied to examine the genomic *P* element. DNA was extracted from ten adults of each population by using Qiagen DNA isolation kit (DNeasy Blood and Tissue Kit, Qiagen, www.qiagen.com ). Genomic DNA from each individual was amplified to determine the size of *P* elements. A single inverted repeat primer (12/2896) AAC ATA AGG TGG TCC CGT CG (31/2877) was used for the *P* element array reaction ( Rasmusson et al. 1993 ). This primer was homologous to the 31 bp terminal inverted repeat of *P* element. To check the amplification from the inverted repeat primer, a second amplification was made with two different primers: 1) the 5' subterminal primer, (35) GCC GAA GCT TAC CGA AGT AT (54) derived sequence inside the 5' inverted terminal repeat of *P* element, and 2) *KP* specific primer, (2577) ATC AAC ATC GAC GTT TCC AC (805), which included the deletion breakpoint in *KP* elements. PCR with this primer combination will only amplify KP elements (see Rasmusson et al. 1993 ) ( Figure 1 ).

PCR was performed in a final volume of 25 µL. For each reaction, 1 µL of genomic DNA was used. Both *P* and *KP* elements were amplified under the following thermal profile. An initial denaturation step at 94° C for 2 min, followed by 30 cycles of 60 sec at 94° C, 60 sec at 60° C, and 90 sec at 72° C following the extension of products at 72° C for 3 min. To determine the size of the *P* elements amplification, products were visualized by agarose gel electrophoresis.

### Statistical analyses

Each isofemale line is characterized by two phenotypes, A and A* crosses scored as the percentage of undeveloped ovaries. Pearson’s test for correlation was performed to evaluate the relationship between altitude and the climatic variables (temperature, humidity, and rainfall) and the *P* activity and repression ability. All tests were done by using SPSS version 17 (IBM, www.ibm.com ).

## Results

To reveal the current status of the P-M system characteristics of wild population of *D. melanogaster* , 88 isofemale lines were examined by GD test ( Table 2 ). M' strains were found to be the most abundant in all local populations examined, and they were predominant overall (84%, 74/88). Of the 88 lines, only one line was found to be P', four lines were M, and nine lines were Q ( Figure 2 ).

**Table 2.**
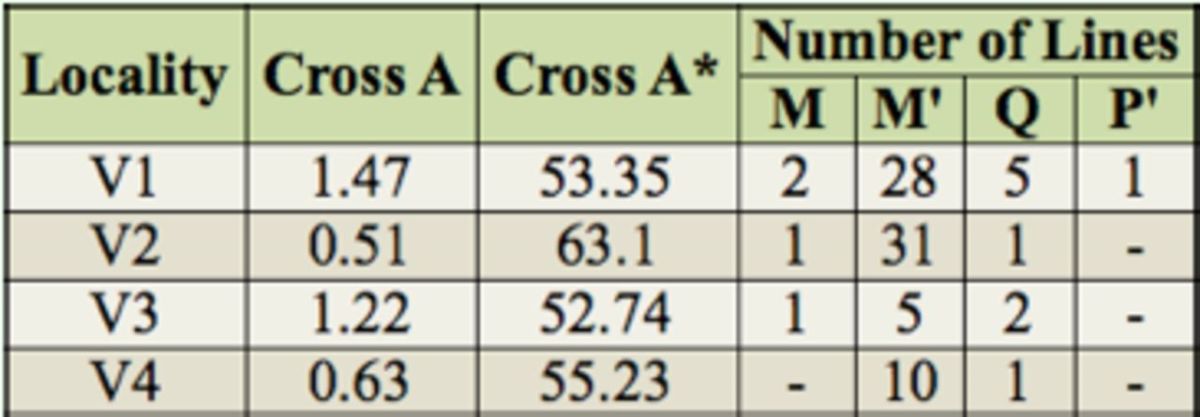
The results of cross A ( *P* activity) and A* ( *P* susceptibility) obtained by using GD test. Each cross is presented as mean of isofemale lines. Cytotypes are classified according to Kidwell (1983) and Quesneville and Anxolabéhère (1998) .

However, there were some variations among phenotypes. The inducing ability of *P* transposition tested in cross A had a variation between isofemale lines, differing from 0 to 25%. The repression ability of all populations tested with cross A* varied between 0 and 100%. The exceptional P' strain ( *V1-37* ) had a high inducing (25%) and repressing (75%) ability. In general, the *P* activity was low in all lines. According to the A cross results, V1 station showed an activity in eight lines between 1.85 and 25%, while the other 28 lines were found to be lacking *P* activity (0%). Similar results were obtained for the second station, V2, as four lines had an activity between 2.78 and 7.14% and the rest of the 29 lines had no *P* activity. For the third station, where the V3 population was sampled, only two lines showed a *P* activity of 2.63 and 7.14%, while the other six lines showed no activity. The activity values obtained for the two lines collected in the last station (V4) were recorded as 1.35% and 5.56%, respectively. The rest of the nine lines were found to have no *P* activity ( Figure 2 ).

The populations were classified in four cytotypes (M, M', Q, and P'). The GD results showed variations among the isofemale lines. Variations in the scope of altitudinal origins and the climatic parameters (temperature, humidity, and rainfall) of the sampling stations were analyzed ( Table 3 ). The correlation between altitude and climatic variables and the GD percentage were analyzed with Pearson’s correlation test. No correlations were detected between the *P* activity (A cross) or the *P* susceptibility (A* cross) and the geoclimatic variables ( Table 3 ).

**Table 3.**
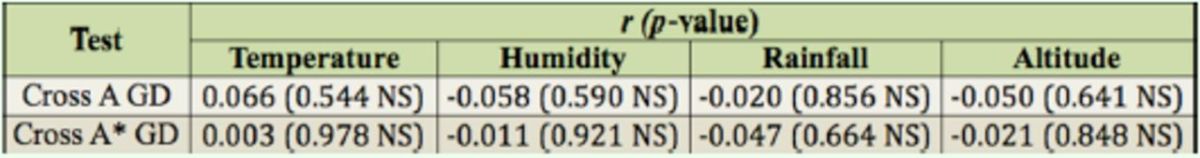
Pearson correlation coefficient ( *r* ) and p values (‘NS’ means ‘non-significant’ at the overall 0.05 level) between altitude and climatic variables with the GD percentage from A and A* cross results.

**Figure 3.**
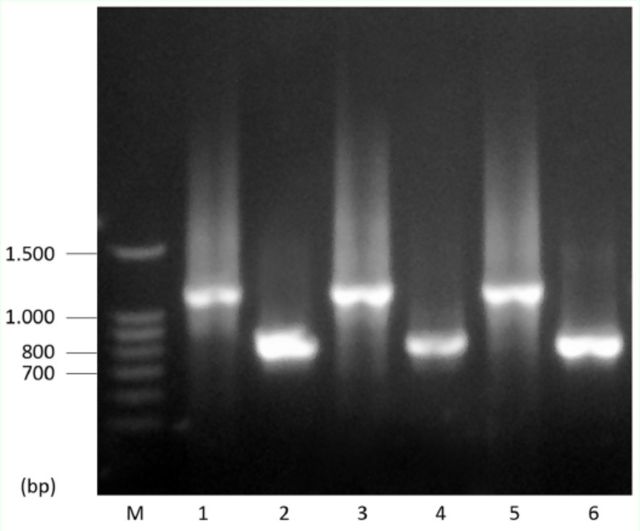
PCR amplification of *P* sequences in strains with isolated *P* elements. Amplifications with the inverted repeat (IR) primer (Lanes 1, 3 and 5) and *KP* specific and 5'-subterminal primers (Lanes 2, 4 and 6). Lanes are as follows: (M) marker lane, (1-2) M' strain, (3-4) Q strain, (5-6) P' strain. High quality figures are available online.

All lines examined had *P* element in their genomes ( Figure 3 ). All phenotypically M lines were, therefore, M' by definition ( Bingham et al. 1982 ). The *KP* elements were the majority in all populations. No other size specific classes were found for P', Q, M, or M' strains. No clear relationship was found between genomic *P* element profiles and P-M phenotypic characteristics.

Samples of 10 adults per isofemale line were tested for the presence of *P* elements by PCR amplification. The existent of genomic *P* elements was examined by two PCRs, one of full-size *P* element and the second of identification of the *KP* elements. The PCR amplification with the inverted repeat primer of the *P* element resulted in a smaller product than 2.9 kb in all samples. The sizes of the all PCR products were approximately 1.15 kb ( Figure 3 ). To check this, a second PCR amplification of the genomic DNA was performed using a primer near the 5' end of the *P* sequence (5' subterminal primer) and a primer including the deletion point in *KP* elements (this primer amplified only the *KP* element) (see Rasmusson et al. 1993 ). In all samples, PCR products were detected to be the same size. These results show that each amplified *P* element was a *KP* element and all P', Q, M', and M strains carried a high copy number of *KP* elements in their genomes. The isolated 1.15 kb *P* element was possibly the same type of *KP* element that is widely distributed in most Eurasian populations of *D. melanogaster* . On the other hand, the PCR results may be an indicator of the absence of full-sized *P* elements or their low copy number in the genome.

## Discussion

The copy numbers of full-sized and defective *P* element in *D. melanogaster* populations and P-M phenotypes vary in worldwide populations. Transposition rates, number of autonomous and non-autonomous copies, environmental effects, history, and population structure could be affected by the relationships between transposable elements and genomes ( Picot et al. 2008 ). Some of the transposable elements’ frequency changes are explained by environmental variables such as mean temperature and mean rainfall (Gonzalez et al. 2010).

*P* activity is affected by both internal and external variables. Environmental stress can lead to an increase of transposon activity ( Capy et al. 2000 ). For instance, it is known that *P* element transposition is accelerated in high temperatures. The results of our study show that a positive but insignificant correlation exists between *P* activity and environmental temperature. With the other environmental factors (humidity and rainfall), *P* activity showed a negative but insignificant relationship. On the other hand, the results of previous work revealed a significant correlation between *P* activity and the mean annual rainfall, suggesting a possibility that rainfall itself might be a stress factor for *D. melanogaster* populations ( Onder and Bozcuk 2012 ). To check these previous results, we investigated the effects of rainfall on the *P* element activity of Firtina Valley populations. The experimental results of the P-M system phenotypes, which were collected from four localities along a transect in Firtina Valley, showed no significant relationship between altitude and climatic variables (mean temperature, mean humidity, and mean rainfall). When compared to the previous data from different parts of Turkey ( Onder and Bozcuk 2012 ), *P* activities were found to be very low. Perhaps the low temperature in this region led to the low *P* activity because of the relationship between high temperature and accelerating *P* mobility in the genome. In this study, 72 lines (82%) without any *P* activity were found. This suggests that rainfall and/or other environmental factors in the Firtina Valley are not inducing *P* element activity. Also, lines collected along the valley from different altitudes did not show wide variation in *P* activity and *P* susceptibility.

Overall, the P-M status distribution of populations showed a predominant M' phenotype, irrespective of geographical origin. Four lines were M, nine lines were Q, and only one isofemale line showed the exceptional P' phenotype. The other 74 lines were M'. These results are quite similar to those of Eurasian populations (Periquet et al. 1989). A theoretical study reported by Quesneville and Anxolabéhère (1998) showed that populations could become any type of strain after *P* element invasion. After invasion, the population can stay stable. But migrations shifting the equilibrium states from P to M may reduce the total number of copies, increase the number of defective *P* elements, decrease the *P* activity, and increase the *P* susceptibility. The equilibrium states are stable for all P-M types, including the exceptional P' type. One of the isofemale lines collected from the coast site Ardesen (V1-37) showed a P' type. Together with the probable P' type observed in four population collected from a distinct region in Turkey ( Onder and Bozcuk 2012 ), this population may support the theoretical study. The observation of a high *P* activity (25%) and *P* susceptibility (75%) in the line V1-37 refers to a certain P' existent in nature.

The molecular findings support that all the tested populations had *KP* elements in their genome. Amplification products of *P* element visualized by agarose gel electrophoresis determined the presence of 1.15 kb *P* elements ( *KP* element) in all populations ( Figure 3 ). These results suggest that the 88 isofemale lines of *D. melanogaster* contained *KP* elements in their genomes. The *KP* elements may have a higher transpose attribution than other *P* element size classes, and their transpositional advantage can make this element predominant in the populations concerned ( Fukui et al. 2008 ). Additionally, the four M strain, 0% dysgenic ovaries in A cross and 100% undeveloped ovaries in A* cross, contained *KP* elements in their genome, which means that these lines are not true M strains and their phenotypes are M' because of the *P* sequences in the genome ( Bingham et al. 1982 ).

For understanding the *P* element dynamics in natural populations, it is necessary to determine the copy number of deleted and full-length *P* elements using Southern blot analyses. Additionally, more molecular data about *P* elements’ size and copy number are needed to understand the exceptional P' populations observed in this study and some other studies, such as Itoh et al. (2001 , 2004).

## Abbreviations

GD: gonadal dysgenesis
